# The Effect of Donor Rat Gender in Mitochondrial Transplantation Therapy of Cisplatin-Induced Toxicity on Rat Renal Proximal Tubular Cells

**DOI:** 10.5812/ijpr-135666

**Published:** 2023-04-08

**Authors:** Abdollah Arjmand, Mehrdad Faizi, Mohsen Rezaei, Jalal Pourahmad

**Affiliations:** 1 Department of Toxicology and Pharmacology, School of Pharmacy, Shahid Beheshti University of Medical Sciences, Tehran, Iran; 2 Department of Toxicology, Faculty of Medical Sciences, Tarbiat Modares University, Tehran, Iran

**Keywords:** Cisplatin, Oxidative Stress, Nephrotoxicity, Mitochondrial Transplantation

## Abstract

**Background:**

Cisplatin-induced nephrotoxicity has been linked to a fundamental mechanism of mitochondrial dysfunction. A treatment called mitochondrial transplantation therapy can be used to replace damaged mitochondria with healthy mitochondria. Mitochondrial-related diseases may benefit from this approach.

**Objectives:**

We investigated the effect of mitochondrial transplantation on cisplatin-induced nephrotoxicity using freshly isolated mitochondria obtained from renal proximal tubular cells (RPTCs).

**Methods:**

Based on our previous findings, we hypothesized that direct exposure of healthy mitochondria to cisplatin-affected RPTCs might improve cytotoxicity markers and restore mitochondrial function. Therefore, the primary objective of this study was to determine whether newly isolated mitochondrial transplantation protected RPTCs from cisplatin-induced cytotoxicity. The supply of exogenous rat kidney mitochondria to cisplatin-affected RPTCs was also a goal of this study to investigate the possibility of gender differences. After the addition of cisplatin (100 µM), rat RPTCs (10^6^ cells/mL) were suspended in Earle’s solution (pH = 7.4) at 37°C for two hours. Freshly isolated mitochondria were extracted at 4°C and diluted in 100 and 200 µg/mL mitochondrial protein.

**Results:**

Statistical analysis revealed that transplantation of healthy mitochondria decreased ROS level, mitochondrial membrane potential (MMP) collapse, MDA level, glutathione depletion, lysosomal membrane damage, and caspase-3 activity induced by cisplatin in rat RPTCs. In addition, our results demonstrated that transplantation of female rat kidney mitochondria has higher protective activity at reducing toxicity parameters than male mitochondria.

**Conclusions:**

The findings reaffirmed that mitochondrial transplantation is a novel, potential, and promising therapeutic strategy for xenobiotic-induced nephrotoxicity.

## 1. Background

Mitochondrial dysfunction is a common and fundamental mechanism for xenobiotic nephrotoxicity, consistent with the common aspect that the mitochondrial membrane and mitochondrial energy-producing capacity are impaired due to the harmful effects of some drugs given to people and the blocking of multiple cell signaling pathways (1). Cisplatin, or cis-diamminedichloroplatinum (II), is a widely used chemotherapy drug that is useful in treating many cancerous tumors, including bladder, cervical, testicular, ovarian, lung, and head, and neck cancers (2, 3). Nevertheless, some studies showed that cisplatin’s critical side effect is dose-related nephrotoxicity (4), which may happen in acute or chronic treatments (5, 6). Cisplatin is excreted primarily by the kidneys and accumulates in the renal cortex. Cell damage occurs in renal proximal tubular cells (RPTCs), including cytoplasmic vacuolation and hydropic damage after cisplatin administration (7). Previous reports also recommended that the nephrotoxicity of cisplatin is related to mitochondrial damage (8), lack of glutathione (GSH) (9), and peroxidation of lipids (10).

Mitotherapy, which involves replacing damaged mitochondria with newly isolated ones, is the most comprehensive and direct method for preventing cisplatin-induced toxicity in RPTCs. Mitochondrial therapy has been suggested to treat mitochondrial-related cytotoxicity, which is feasible (11, 12). Recent studies have recommended that freshly isolated mitochondria can enter mammalian cells by incubation and protect receiving cells against cytotoxicity initiated by mitochondrial injury (13-15). The isolated mitochondria from female animals are more resistant to stress conditions and give better performance and efficiency in harmful conditions (16, 17). As a result.

## 2. Objectives

This study aimed to determine if mitochondrial transplantation protected RPTCs from cisplatin-induced cytotoxicity and to examine the effect of gender differences in rat mitochondrial donors on the effectiveness of mitochondrial transplantation therapy against RPTCs affected by cisplatin.

## 3. Methods

### 3.1. Animals

In all of the studies, male and female Wistar rats weighing between 250 and 300 grams were used. They were fed a standard diet and drank water. Under controlled conditions of temperature (20 - 25°C), humidity (70 - 80%), and the usual light-dark cycle (12:12 hours), they were kept in different folds. During the experiments, every effort was made to minimize the suffering of the animals. The protocols for the animal experiments were approved by the Animal Ethics Committee of Shahid Beheshti University of Medical Sciences (IR.SBMU.PHARMACY.REC. 1399.050).

### 3.2. Chemicals

MilliporeSigma in St. Louis, Missouri, provided cis-dichlorodiammineplatinum (II), Acridine Orange, 2′, 7′-dichlorofluorescin diacetate (DCFH-DA), Hanks’ Balanced Salt Solution (HBSS), ortho-phthalaldehyde (OPA), cytochalasin D, 5-(N-Ethyl-N-isopropyl)amiloride (EIPA), methyl-β-cyclodextrin, Tris-HCl, D-mannitol, ketamine, xylazine and trichloroacetic acid (TCA), 3-(4,5-dimethylthiazol2-yl)-2,5-diphenyltetrazolium bromide (MTT), rhodamine 123, Coomassie blue, collagenase type II, dimethylsulphoxide (DMSO), N-(2 hydroxyethyl)piperazine-N′- (2-ethane sulfonic acid) (HEPES). The highest commercial quality was present in all of the other chemicals.

### 3.3. Preparation and Isolation of RPTCs Cells

In order to isolate RPTCs, the enzymatic procedures were modified (18, 19). Ketamine (40 mg/kg) and xylazine (10 mg/kg) were used to anesthetize Wistar rats weighing 250 and 300 g. After harvesting the kidneys, cervical dislocation was used to scarify the animals. Ca^2+^-free HBSS (Hank’s balanced salt solution) with 0.5 mM EGTA was used to perfuse the kidneys. After that, the samples were digested in an HBSS solution containing 0.05% collagenase type II, 4 mM CaCl_2_, and 1% penicillin/streptomycin. The kidneys’ cortical segments were then decapsulated and dissected to obtain tubules of proximal cells mechanically separated by continuous filtration at 120 µm and 60 µm mesh, respectively, and fragments ranging in thickness from 0.5 to 1 mm. Pellets were made from the washed and extracted RPTCs. After that, the isolated cells were resuspended in Earl’s solution (pH = 7.4) at a concentration of 1 10^6^ cells per milliliter, placed in round-bottomed containers, circulated in a 37°C water bath, and added to 28 mM HEPES that was surrounded by 5% CO_2_, 10% O_2_, and 85% N_2_.

### 3.4. Assay for Cell Viability

The release of lactate dehydrogenase (LDH) from cells indicates cytotoxicity and cell death. The LDH activity was measured using the LDH kit from MilliporeSigma (St. Louis, Missouri) briefly, 10 µL of the sample was combined with 1 mL of the indicator at 37°C, and the Beckman DU-7 spectrophotometer recorded the absorbance at 30 s intervals for four minutes. Using a relative factor, the sample’s mean change in absorbance at 340 nm is converted into units of enzyme activity. For each treatment group, the LDH activity was reported as µM substrate/min/L. (20).

### 3.5. Measurement of Mitochondrial Activity, Normalization, and Isolation

Active and new mitochondria were extracted from the kidney of a Wistar rat using differential ultracentrifugation (Hettich, Universal 320R, Germany) (21, 22). In a cold isolation solution containing 75 mM sucrose, 0.2 mM EDTA, and 0.225M D-mannitol, the kidneys of a rat were removed and chopped with scissors. To remove intact cells and nuclei, the kidneys were homogenized with a glass homogenizer and centrifuged at 1000 g for ten minutes at 4°C. After that, the supernatant was treated with 250 µL of BSA solution, which was then continuously filtered through 40 and 5 µm meshes, respectively. For ten minutes, the supernatant was centrifuged at 10,000 g. The mitochondrial fraction, or lower layer, was resuspended in the isolation solution and centrifuged twice for 10 minutes at 10,000 g. It was then suspended in a homogenization buffer containing 0.05 M Tris-HCl, 2.0 mM MgCl_2_, 20mM KCl, 0.25 M sucrose, and 1.0 mM Na_2_HPO_4_ (PH = 7.4) at 4°C. The protein concentrations of the isolated mitochondria were determined using the Coomas (23). For each subsequent mitochondrial assay, mitochondrial samples (0.5 mg mitochondrial protein/mL) were used in the normalization procedure. An ELISA reader (Tecan, Rainbow Thermo, Austria) was used to measure the reduction of MTT at the absorbance of 570 nm to analyze mitochondrial succinate dehydrogenase (24).

The mitochondrial membrane potential was determined by measuring mitochondria’s Rhodamine123 (Rh123) uptake. In the mitochondrial membrane potential (MMP) assay solution, 0.5 mg protein/mL of the mitochondrial sample was incubated with 10 µM of Rh123. After that, fluorescence at excitation = 490 nm and emission = 535 nm was measured with a spectrofluorometer (Shimadzu RF5000U, Japan) (25).

### 3.6. Study Design

Rat RPTCs (106 cells/mL) were suspended in Earle’s solution (PH = 7.4) at 37°C for two hours following the addition of cisplatin (100 µM). In addition, mitochondria for transplantation were isolated from the kidney at 4°C, and the optimal dose was chosen based on our previous research. In a water bath maintained at 37°C, mitochondrial-supplemented media was used to replace the media on RPTCs and was left on the cells for 1, 2, 3, and 4 hours.

### 3.7. ROS Measurement in the RPTCs

7′-Dichlorofluorescin diacetate (1.6 µM) was added to the cells to examine the rate of ROS production in the RPTCs following mitochondrial transplantation (mitotherapy). 7′-Dichlorofluorescin diacetate was hydrolyzed into non-fluorescent DCFH after entering the RPTCs. The latter then reacted with ROS to produce highly fluorescent DCF. A spectrofluorometer (Shimadzu RF5000U, Japan) with excitation and emission wavelengths of 500 nm and 520 nm was used to measure the fluorescence intensity of DCF, a ROS product. The fluorescence intensity units per 106 cells were used to report the results (9).

### 3.8. Analysis of the MMP in the RPTCs

The uptake of Rhodamine123 (Rh123), a cationic fluorescent dye, was utilized for determining the MMP. Centrifugation was used to separate 0.5 mL of the RPTCs suspension from the medium solution. The pellet was then resuspended in 2 milliliters of Rh123 (1.5 µM) containing medium and incubated for ten minutes at 37 degrees Celsius. Using a fluorescence spectrofluorometer (Shimadzu RF5000U, Japan) with an excitation wavelength of 490 nm and an emission wavelength of 520 nm, the amount of Rh123 in the RPT cell suspension was measured. The difference in Rh123 fluorescence between the control and treatment groups was used to calculate the mitochondrial ability to absorb Rh123 (26).

### 3.9. Test for Lysosomal Membrane Damage in RPTCs

The fluorescent dye Acridine Orange redistribution was used to measure the lysosomal membrane stability in RPTCs. By centrifugation at 1000 g for one minute, the samples (0.5 mL) of cell suspension that had previously been incubated with Acridine Orange (5 µM) were separated from the medium. After that, the dye was removed from the media by twice resuspending the pellet in 2 milliliters of the new incubation medium. Following that, a fluorescence measurement with a spectrofluorometer (Shimadzu RF5000U, Japan) at an excitation wavelength of 495 nm and an emission wavelength of 530 nm was used to measure the redistribution of Acridine Orange in the cell suspensions (27).

### 3.10. Lipid Peroxidation Measurement in the RPTCs

Malondialdehyde (MDA) levels were measured to assess lipid peroxidation (LPO), an indicator of oxidative stress. An ELISA reader (Tecan, Rainbow Thermo, Austria) was used to measure the supernatant’s absorbance at 532 nm to determine the MDA level in the samples (28).

### 3.11. The RPTCs GSH and GSSG Measurements

After adding TCA 10% (0.5 mL) to the RPTCs, they were centrifuged for two minutes at 11,000 g. After that, 4.5 mL of EDTA buffer was used to dilute 0.5 mL of the supernatant. After that, 100 µL of ortho-phthalaldehyde solution (OPA) and 1 mL of the supernatant that had been diluted were added to 2.8 mL of phosphate-EDTA buffer. Finally, fluorescence intensity was measured using a spectrofluorometer (Shimadzu RF5000U, Japan) at the excitation wavelength of 350 nm and emission wavelength of 420 nm following a 15-minute incubation at 25°C (29).

### 3.12. Test for Caspase-3 Activity in the RPTCs

Caspase-3 activity was measured with a colorimetric caspase-3 assay kit. The peptide substrate (ACDEVD-pNA) is hydrolyzed by caspase-3 in this assay. At 405 nm, the absorbance of pNA was measured (30).

### 3.13. Screening of Cellular Uptake Mechanism of Isolated Mitochondria

To examine the mechanism of cellular uptake of isolated mitochondria, Cisplatin-treated RPTCs (10^6^ cells/mL) were pre-incubated with cytochalasin D (10 µM), an actin-dependent endocytosis inhibitor (31), 5-(N-Ethyl-N-isopropyl) amiloride (EIPA) as a macropinocytosis inhibitor, at a concentration of 100 µM (32), and methyl-cyclodextrin (1 mM) as an endocytosis inhibitor dependent on clathrin (33), in distinct flasks for thirty minutes. Afterward, each flask was co-incubated for four hours at 37°C and 5% CO_2_ with 200 µg protein/mL of isolated mitochondria. The evaluation of the protective effects of mitochondrial transplantation on cytotoxicity parameters, including LDH release, ROS formation, caspase-3 activities, and ATP content, concluded the study. The luciferase enzyme was used to evaluate the amount of ATP (34).

### 3.14. Statistical Analysis

The data were presented as mean ± SD. One-way or two-way analysis of variance (ANOVA) followed by appropriate post hoc tests were used for statistical analysis of each result. The normality test was the Shapiro-Wilk test. P < 0.05 was selected as the minimal level of significance. This study used GraphPad Prism 9 (GraphPad, La Jolla, CA, USA) for statistical analysis and graphic design.

## 4. Results

### 4.1. Function Assay of Freshly Isolated Mitochondria in Mitotherapy

**Figure 1.**
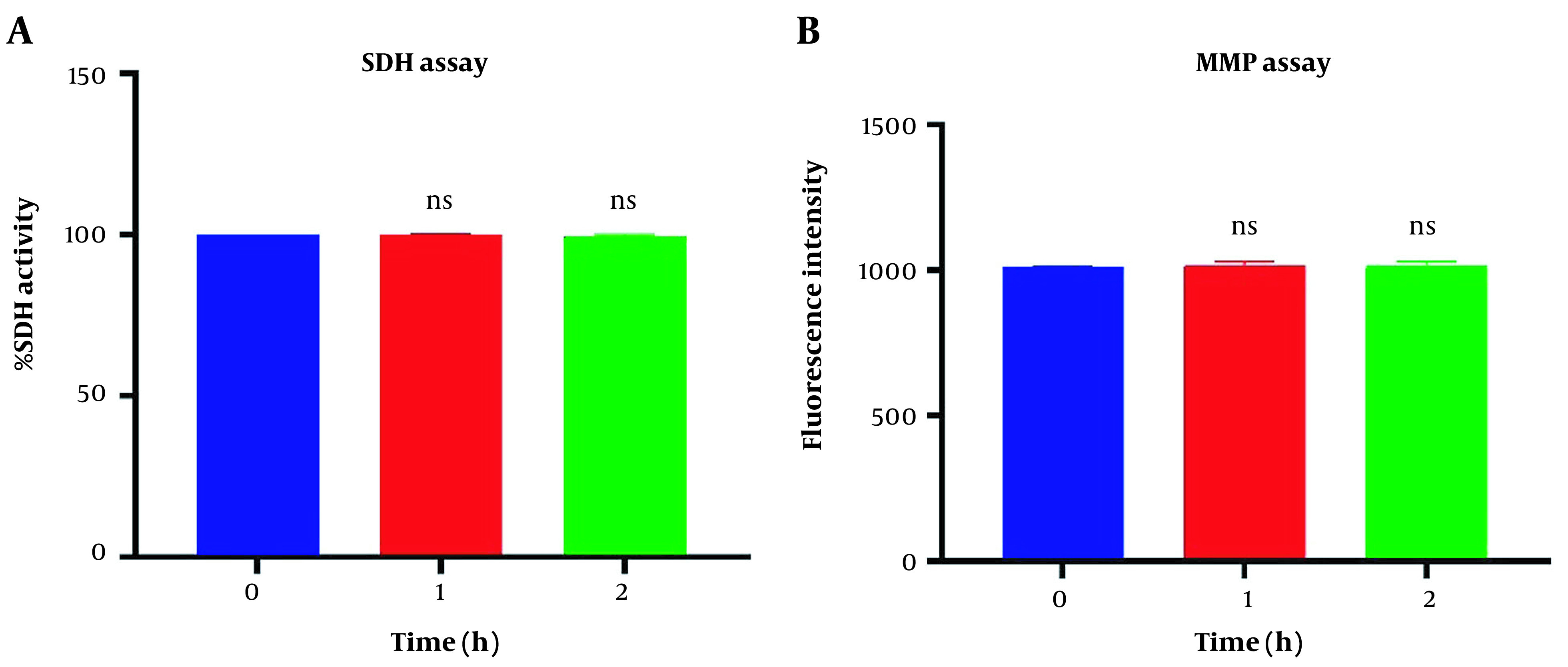
Mitochondrial health assurance assays. Evaluation of succinate dehydrogenase (A) and potential of mitochondrial membrane (B) of isolated mitochondria. SDH activity was measured through 3-(4,5-dimethylthiazol2-yl)-2,5-diphenyltetrazolium bromide (MTT) dye following two-hour incubation. Mitochondrial membrane potential (MMP) was measured by using Rh123 following two-hour incubation. Values were demonstrated as mean ± SD (n = 3). ns. No marked difference vs. zero time in the control group (P < 0.05).

Figure 1A revealed no significant difference in succinate dehydrogenase activity following 1 and 2h of incubation of the freshly isolated mitochondria suspension compared to the control group (P > 0.05). Similar data were observed in MMP assay in Figure 1B. Our data confirmed a non-significant difference in MMP decline following one and two-hour incubation in respiratory buffer media compared to its zero time (P > 0.05).

### 4.2. The Effect of Mitochondrial Transplantation on Viability Assay

**Figure 2.**
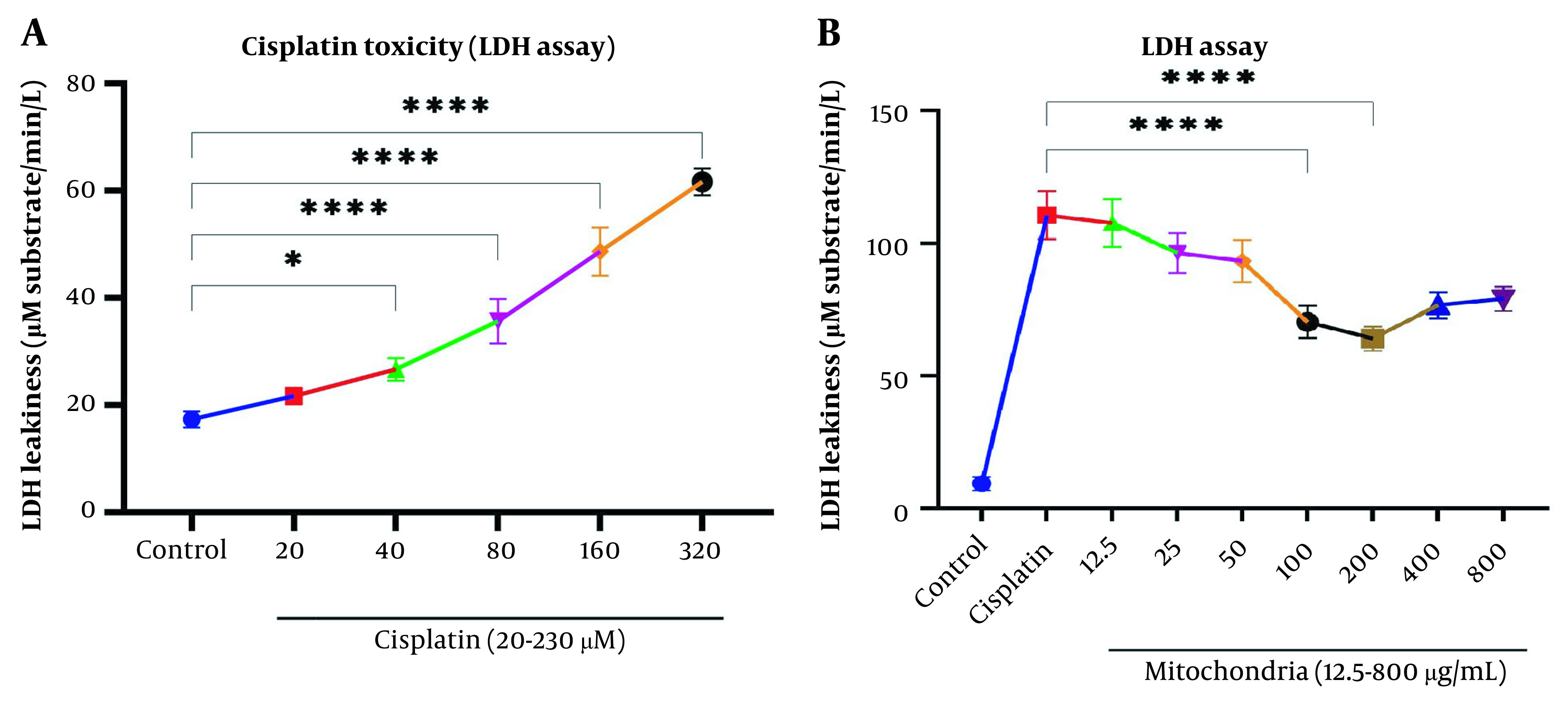
Evaluation of cytotoxicity lactate dehydrogenase (LDH). A, Cisplatin toxicity on renal proximal tubular cells (RPTCs) at different concentrations; B, Prevention of LDH leakiness on RPTCs with different concentrations of freshly isolated mitochondria. Values were presented as mean ± SD (n = 3). ****Significant difference vs. indicated groups (P < 0.0001). *Significant difference vs. the control group (P < 0.05).

**Figure 3.**
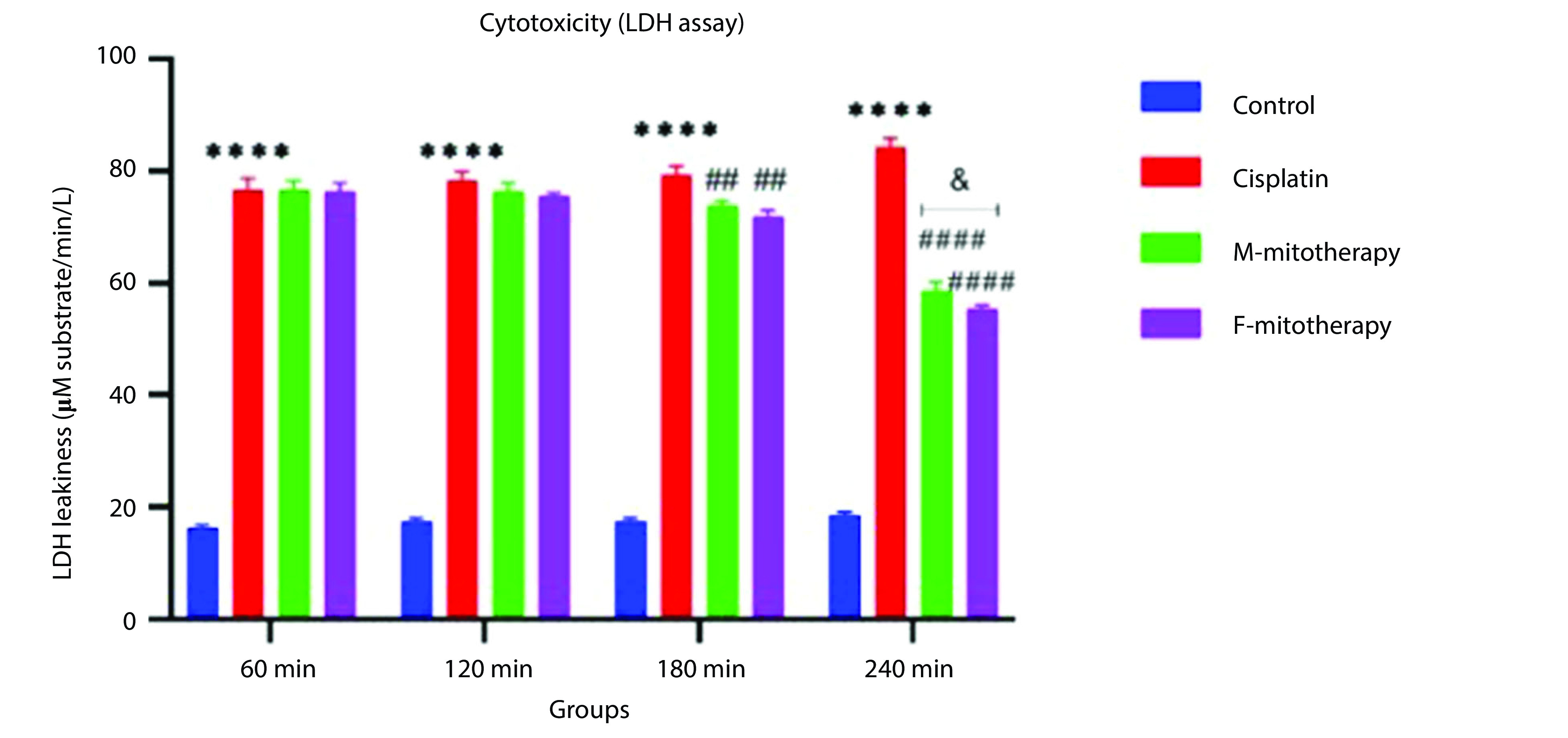
Effect of mitochondrial transplantation on cisplatin-induced lactate dehydrogenase (LDH) leakiness. Prevention of LDH leakiness on renal proximal tubular cells (RPTCs) at different times by female and male mitochondrial transplantation (200 µg/mL). Values are expressed as the mean of three separate experiments (± SD). ****Significant difference vs. control RPTCs (P < 0.0001). ####Significant difference vs. cisplatin group (P < 0.0001). ##Significant difference vs. cisplatin group (P < 0.01). & significant difference vs. M-mitotherapy group (P < 0.05).

Our data showed a significant rise in LDH release following Cisplatin exposure (40, 80, 160, and 320 µM) in RPTCs following two-hour incubation (P < 0.05; Figure 2A). Besides, co-incubation of freshly isolated mitochondria in cisplatin-treated groups prevented cisplatin-induced cytotoxicity (LDH leakage) at low-concentration isolated mitochondria (L-mitotherapy) and high concentration (H-mitotherapy), which was selected based on our previous study (Figure 2B). As shown in Figure 3, mitochondrial transplantation (200 µg/mL protein) could decrease LDH leakiness in the cisplatin-exposed RPTCs after 240 min (4h) co-incubation. Both female and male rat mitochondria decreased LDH leakiness after four-hour co-incubation in RPTCs; however, the female mitochondria showed a better preventive effect (P < 0.01).

### 4.3. The Effect of Mitochondrial Transplantation on ROS Level

**Figure 4.**
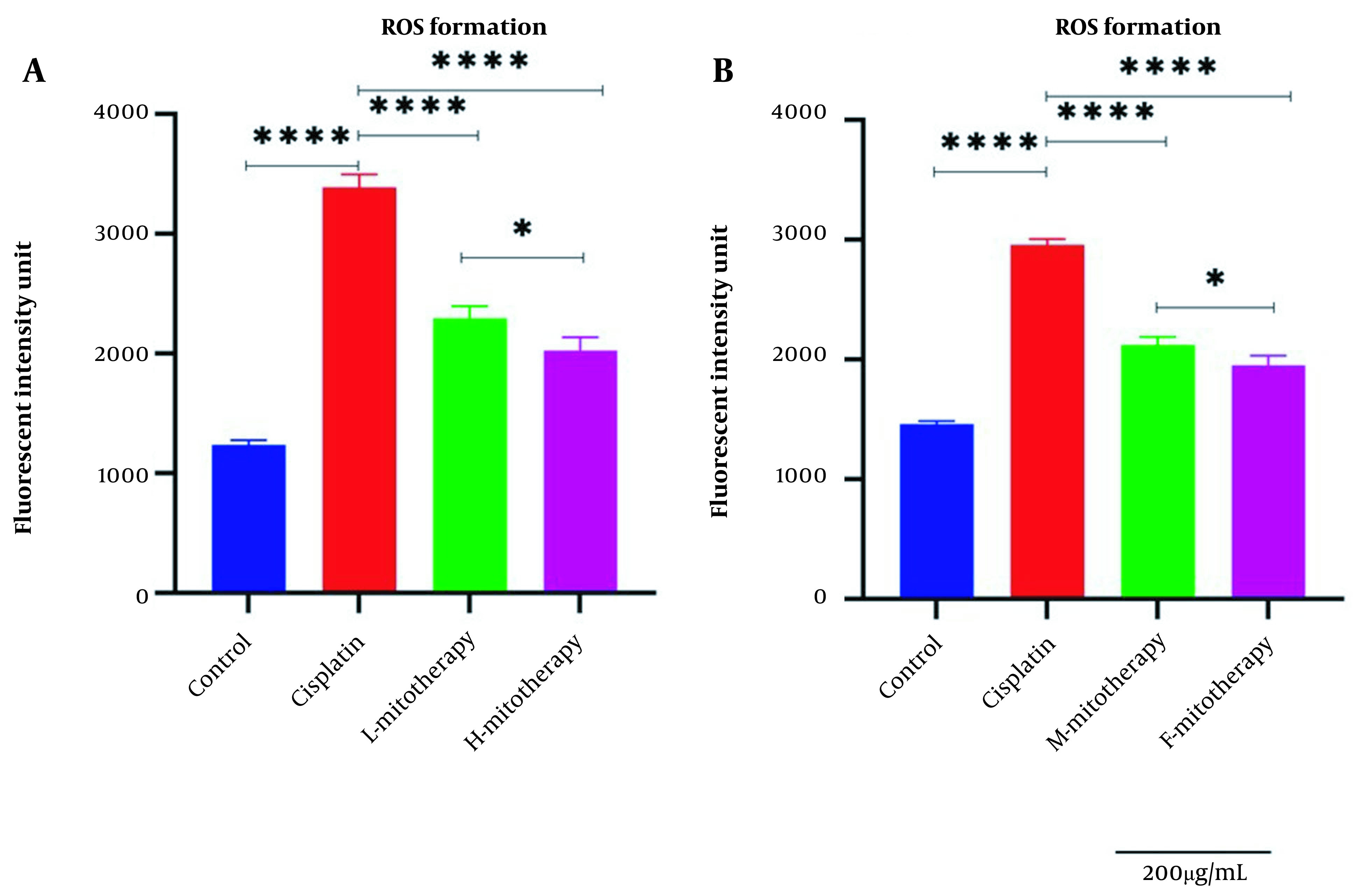
Effect of mitochondrial transplantation on cisplatin-induced ROS production. Evaluation of low and high mitochondria administration (A) and the difference between female and male mitochondria administration (B). Values were presented as mean ± SD (n = 3). **** (P < 0.0001) significant difference vs. indicated groups. * Significant difference between two mitotherapy groups (P < 0.05).

Our results showed a significant rise in ROS levels following the treatment of isolated mitochondria with cisplatin compared to control groups (P < 0.0001; Figure 4). However, after mitochondrial transplantation, ROS levels were significantly reduced in cisplatin-treated cells (P < 0.0001). These results suggested that the transplantation of mitochondria could rescue the RPTCs from oxidative injury caused by cisplatin. As shown in Figure 4B, both the female and male rat mitochondria reduced ROS levels after four-hour co-incubation with RPTCs. However, female mitochondria showed a better preventive effect (P < 0.01).

### 4.4. The Effect of Mitochondrial Transplantation on MMP Assay

**Figure 5.**
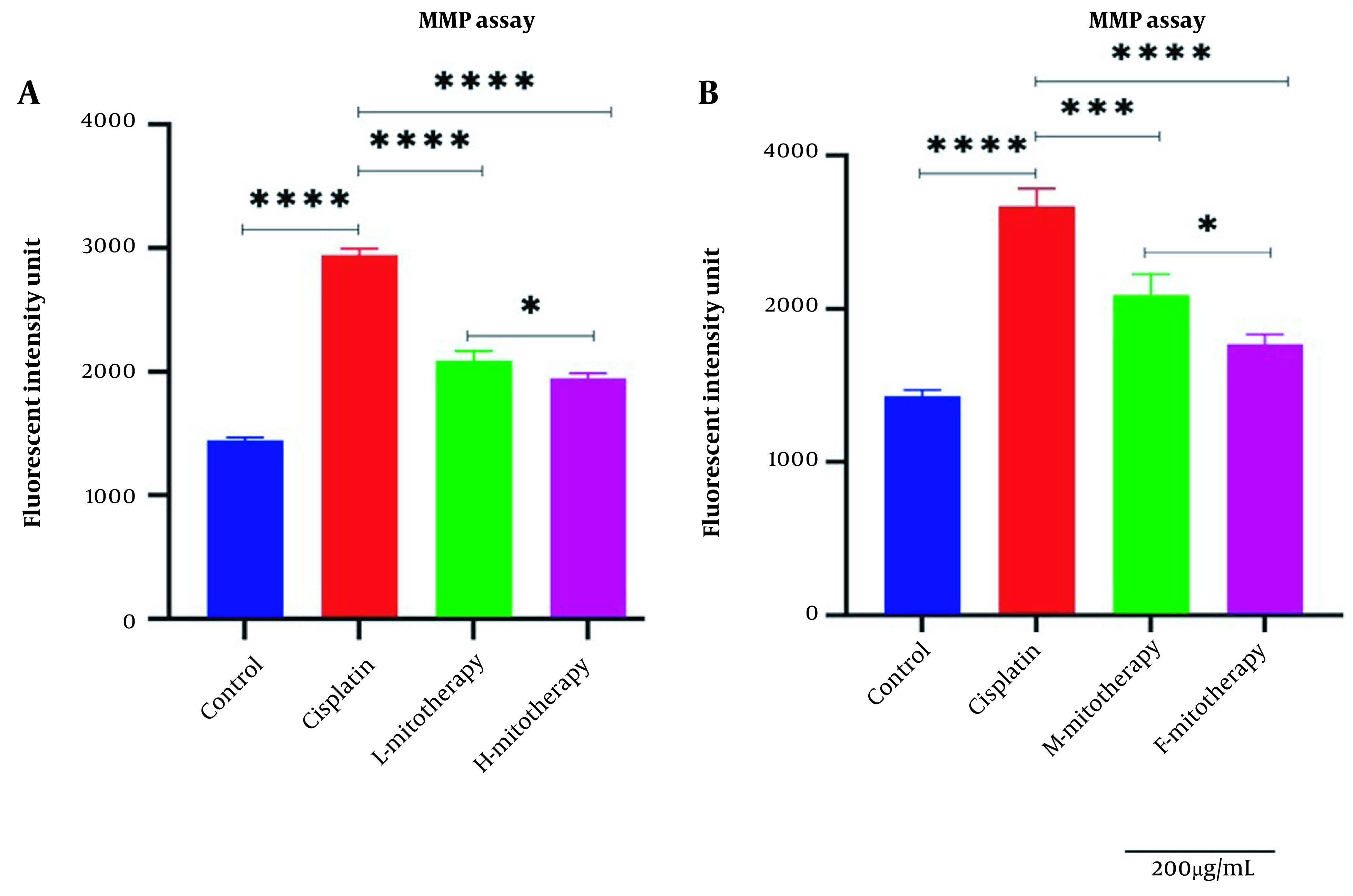
Effect of mitochondrial transplantation on cisplatin-induced Mitochondrial membrane potential (MMP) collapse. Evaluation of low and high mitochondria administration (A) and the difference between female and male mitochondria administration (B). Values were presented as mean ± SD (n = 3). **** (P < 0.0001). *** (P < 0.001). Significant differences vs. indicated groups. * Significant difference between two mitotherapy groups (P < 0.05).

Figure 5A-B revealed a rapid decline in the MMP in cisplatin-treated isolated mitochondria. In addition, mitochondrial transplantation restored MMP decline, the major and direct reason for cisplatin-induced mitochondrial damage. The female mitochondria (F-mitotherapy) and the high mitochondrial concentration (H-mitotherapy) showed more effectiveness than the male mitochondria and low concentration mitochondrial administration (L-mitotherapy), respectively.

### 4.5. The Effect of Mitochondrial Transplantation on Lysosomal Membrane Integrity

**Figure 6.**
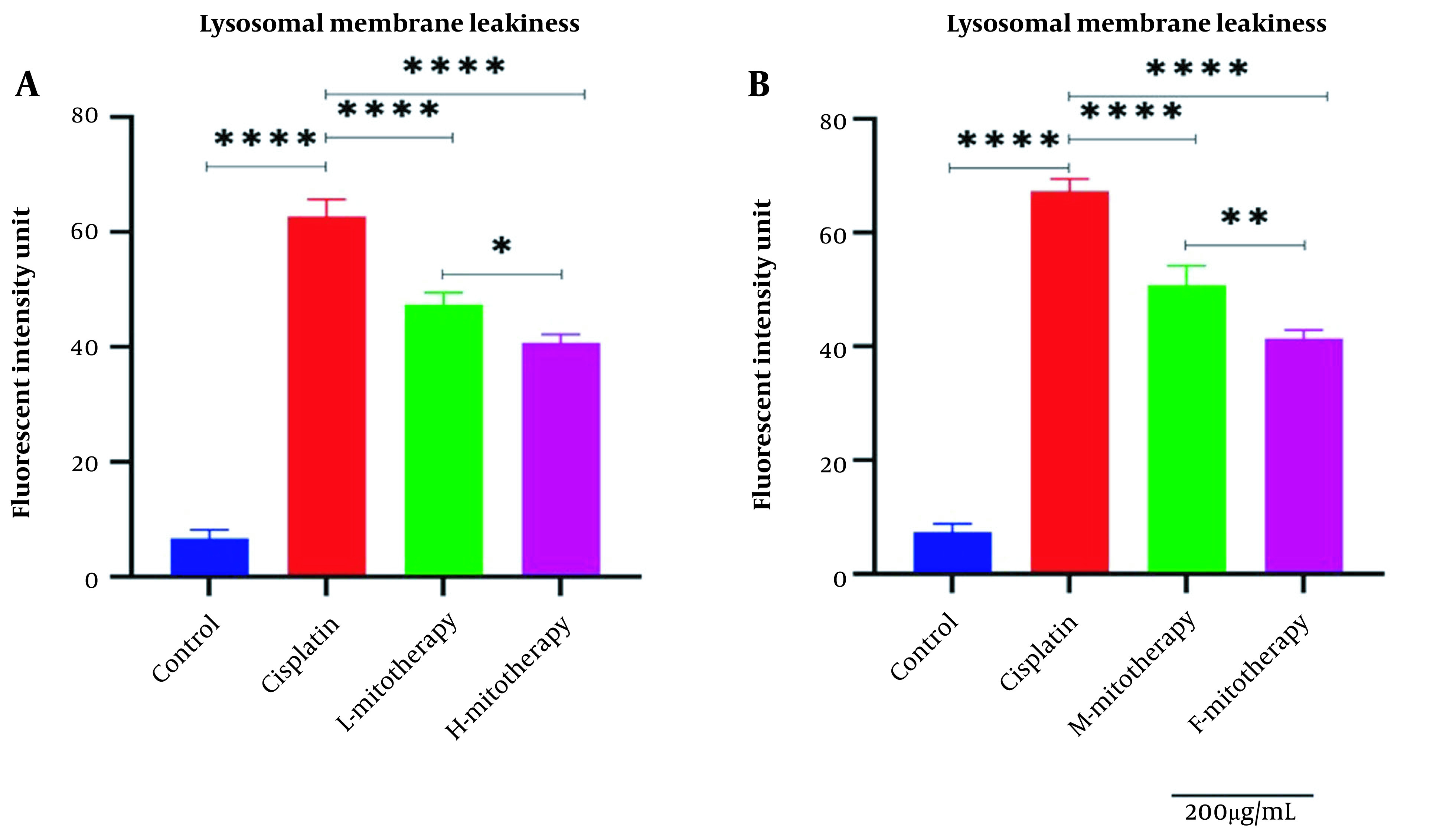
Effect of mitochondrial transplantation on lysosomal membrane leakiness. Comparison of low and high mitochondria administration (A) and the difference between male and female mitochondria administration (B). Values were presented as mean ± SD (n = 3). **** Significant differences vs. indicated groups (P < 0.0001). ** Significant difference between two mitotherapy groups (P < 0.01). * Significant difference between two mitotherapy groups (P < 0.05).

Figure 6 showed that Cisplatin-induced lysosomal membrane damage was inhibited by mitochondrial transplantation in high and low concentrations. As shown in Figure 6B, both female and male rat mitochondria corrected lysosomal membrane integrity after four-hour co-incubation with the RPTCs. However, the female mitochondria showed a better preventive effect (P < 0.01).

### 4.6. The Effect of Mitochondrial Transplantation on Lipid Peroxidation

**Figure 7.**
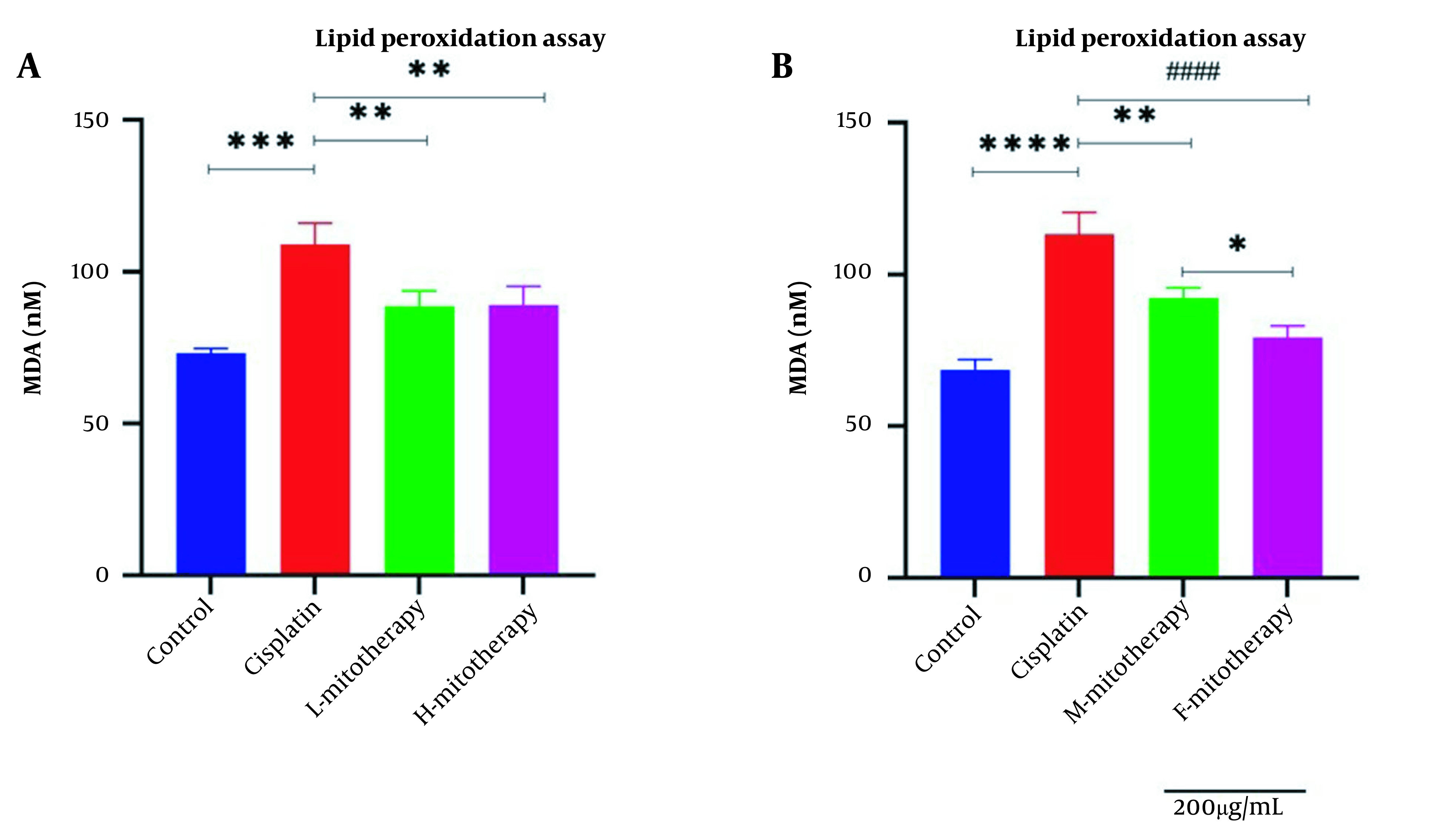
Effect of mitochondrial transplantation on lipid peroxidation (LPO). Comparison of low and high mitochondria administration (A) and the difference between male and female mitochondria administration (B). Values were presented as mean ± SD (n = 3). **** Significant difference vs. control RPTCs (P < 0.0001). #### Significant difference vs. cisplatin-treated RPTCs (P < 0.0001). *** Significant difference vs. control RPTCs (P < 0.001). ** Significant difference vs. cisplatin-treated RPTCs (P < 0.01). * Significant difference between two mitotherapy groups (P < 0.05).

Figure 7 revealed a rise in MDA level following cisplatin incubation in different doses in the RPTCs. Furthermore, mitochondrial transplantation significantly decreased the MDA level of cisplatin exposure. Furthermore, no significant difference was observed between exposure to low and high mitochondrial concentrations. Besides, the isolated female mitochondria revealed a better protective effect than male isolated mitochondria extracted from male rat kidneys (Figure 7A and B; P < 0.01).

### 4.7. The Effect of Mitochondrial Transplantation on GSH Level

**Figure 8.**
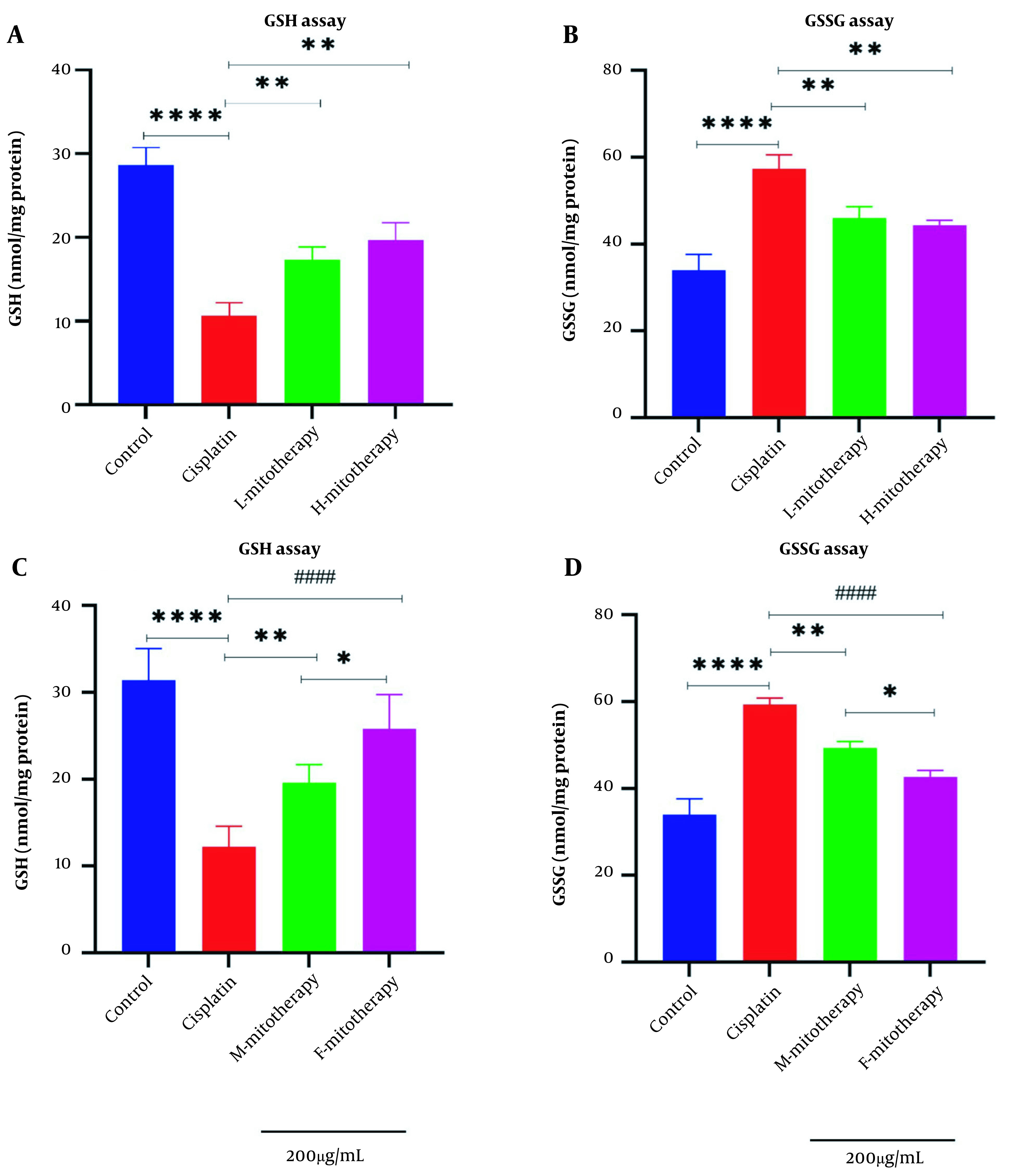
Mitochondrial transplantation effect on glutathione (GSH) and GSSG. lack of glutathione determination (A, C) and GSSG determination (B, D) in renal proximal tubular cells (RPTCs). Renal proximal tubular cells were treated with cisplatin, and freshly isolated mitochondria were added. Values were shown as mean ± SD (n = 3). **** Crucial difference vs. control RPTCs (P < 0.0001). #### Crucial difference vs. cisplatin-treated RPTCs (P < 0.0001). ** Crucial difference vs. cisplatin-treated RPTCs (P < 0.01). * Crucial difference between two mitotherapy groups (P < 0.05).

Results of this study showed that the exposure of the freshly isolated mitochondria with cisplatin exposure could increase the GSH level (Figure 8A) and decrease the GSSG level (Figure 8B) in RPTCs following 4 hours of co-incubation. Besides, there was a significant rise in GSH levels in mitochondrial transplantation + cisplatin groups compared with the cisplatin-treated groups (P < 0.01). In addition, our data revealed that the female isolated mitochondria showed a better protective effect compared to male isolated mitochondria (Figure 8C-D; P < 0.05).

### 4.8. The Effect of Mitochondrial Transplantation on Caspase-3 Activity

**Figure 9.**
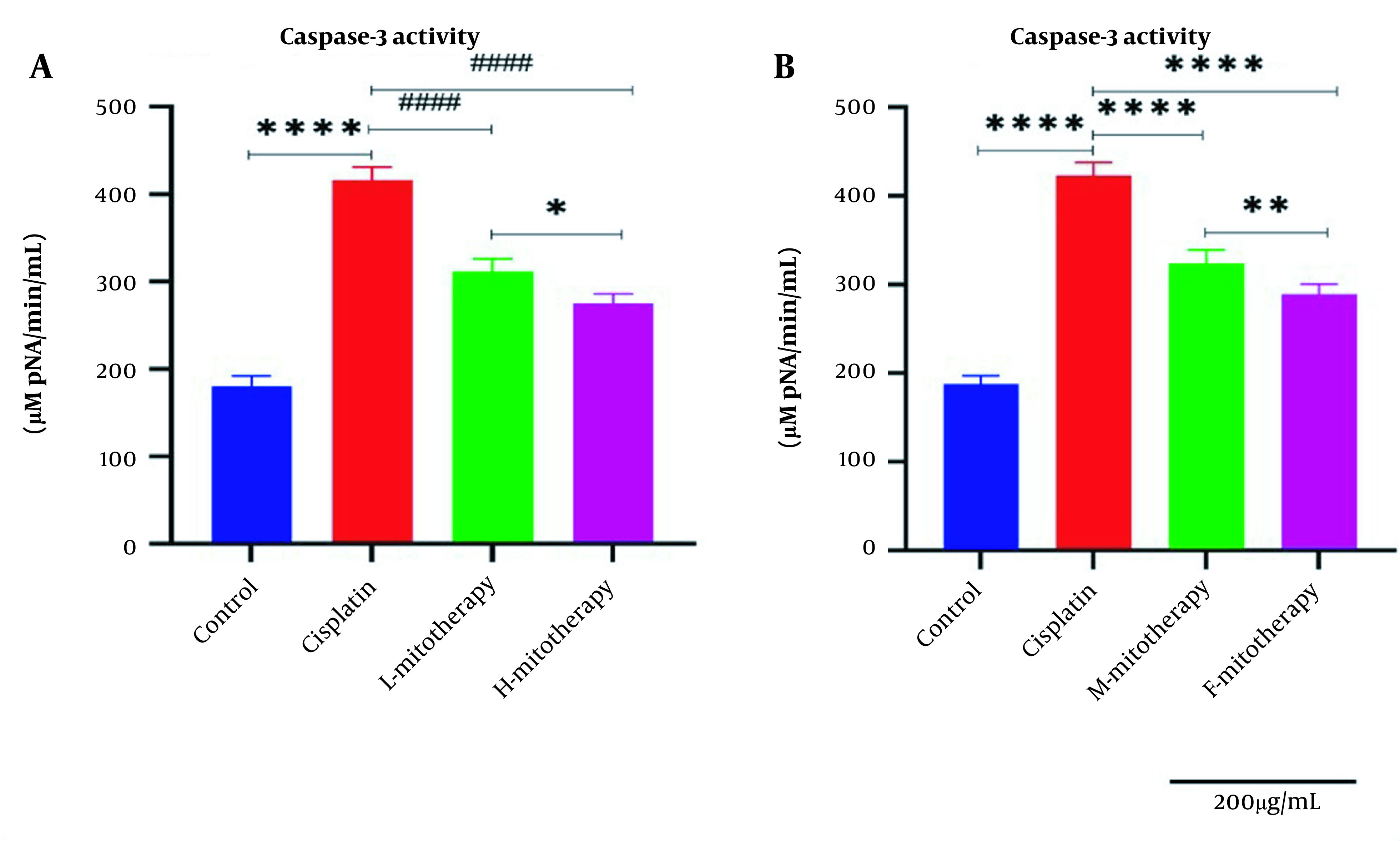
Effect of mitochondrial transplantation on caspase‐3 activity in renal proximal tubular cells (RPTCs) affected by cisplatin. The caspase‐3 activity was lower in the control group, and in the cisplatin group significantly increased. The caspase-3 activity was suppressed in all mitotherapy groups. Values were presented as mean ± SD (n = 3). **** Significant difference vs. control RPTCs (P < 0.0001). #### Significant difference vs. cisplatin-treated RPTCs (P < 0.0001). ** Significant difference.

Our data showed that cisplatin-treated RPTCs following two hours of exposure caused a significant rise in caspase-3 activity (Figure 9). Figure 9A showed that transplantation of freshly isolated mitochondria at both concentrations (L-mitotherapy and H-mitotherapy) caused a significant decrease in caspase-3 activity as a final mediator of apoptosis. Furthermore, the female mitochondria showed a higher protective effect compared to male isolated mitochondria (Figure 9B).

### 4.9. The Effect of Endocytosis in the Internalization of the Isolated Mitochondria

**Figure 10.**
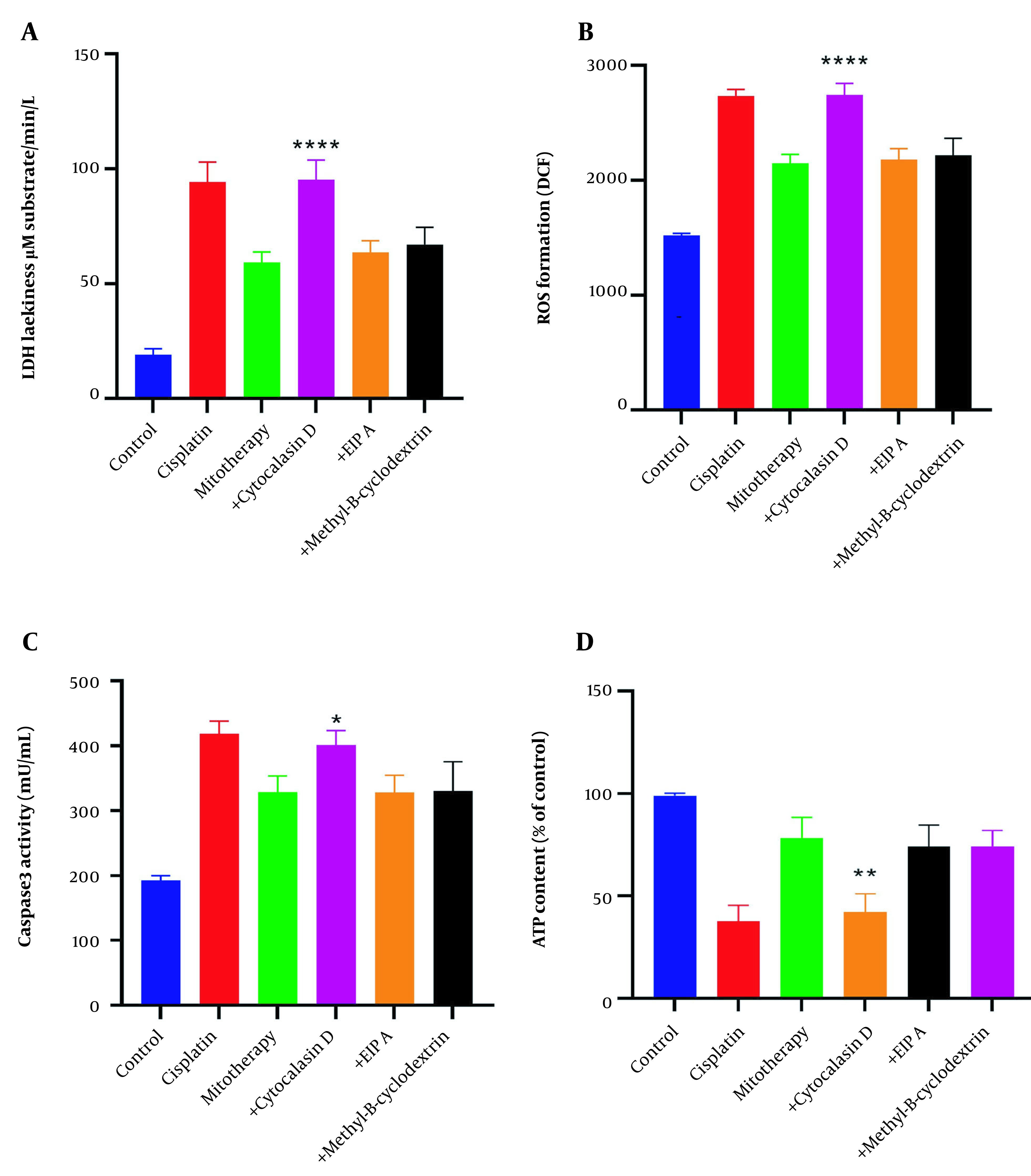
Inhibition of mitochondrial transplantation functions by cytochalasin D. Pre-incubation of renal proximal tubular cells (RPTCs) with cytochalasin D significantly inhibits the effectiveness of mitochondrial transplantation. Lactate dehydrogenase (LDH) assay (A), ROS formation (B), caspase-3 activity (C), and ATP content (D). Values were presented as mean ± SD (n = 3). **** Significant difference vs. mitotherapy group (P < 0.0001). ** Significant difference vs. mitotherapy group (P < 0.01). * Significant difference vs. mitotherapy group (P < 0.05).

The protective effects of mitochondrial transplantation in the RPTCs were significantly inhibited following pre-incubation with cytochalasin D (P < 0.05). As shown in Figure 10A-D, LDH release, ROS formation, caspase-3 activities, and ATP content were only affected following pre-incubation with cytochalasin D. These data demonstrate that actin-dependent endocytosis is involved in the internalization of the mitochondria into the RPTCs.

## 5. Discussion

Mitochondrial dysfunction is a major mechanism in cisplatin-induced nephrotoxicity. Therefore, trying to decrease this dysfunction is an important aim in the reduction of this major toxicity. Our research aimed to suppress this restriction by transplanting the fresh mitochondria extracted from rat kidney homogenate using the ultracentrifugation technique into cisplatin-affected cells. This technique permits mitochondria to work as entire and healthy organelles to recover all cisplatin-induced parameters in the RPTCs (35). For this aim, freshly isolated mitochondria were applied to treat the cisplatin-induced nephrotoxicity. Previous studies showed that Kupffer cells in primary culture could rapidly bind and uptake liver mitochondria which were added to culture media by endocytosis (36). In cells, mitochondria are the most important for energy production (37). Furthermore, mitochondrial transplantation could be considered as a new therapeutic strategy (38).

Cisplatin, a platinum chemotherapeutic medicine prescribed for a series of malignancies, is a cytotoxic agent with clinically significant potential for nephrotoxicity (39). Besides, the nephrotoxicity of cisplatin displays the pathological alterations (40) and biochemical changes following cisplatin-induced nephrotoxicities such as rise in ROS level (41), an increase in MDA (42), cytochrome c release (43, 44), and decline in glutathione level (45). Therefore, oxidative stress is supposed to play a key role in cisplatin-induced nephrotoxicity, which causes cell death signaling and considerable depletion of antioxidant defense molecules (46, 47).

Our recent study indicates that cisplatin can quickly increase ROS generation in the RPTCs following its incubation. Moreover, our results suggest that the cytotoxicity of cisplatin is directly related to an increase in ROS level, which may play an important role in the underlying mechanism of cisplatin-induced kidney damage. In addition, mitotherapy could inhibit cisplatin-induced toxicity, significantly affecting ROS level and MMP collapse following cisplatin exposure in RPTCs at high or even low mitochondrial concentrations. The recent data showed that mitochondrial transplantation positively impacts glutathione content, restoring cell viability.

For this reason, adding freshly isolated mitochondria to the cisplatin-affected RPTCs will be a possible strategy for increasing ATP generation and decreasing cell injury. Results of this investigation showed that ATP decline and MMP collapse cause the release of mitochondrial apoptosis-inducing factors into the cytosol, resulting in caspase-3 activation, the final apoptosis mediator. Furthermore, the main pathway for activation of caspase-3 following incubation of RPTCs with cisplatin is related to the leak of cytochrome c from mitochondria after the MPT opening through the mitochondrial ROS transmission into the lysosomes and the production of lysosomal radicals. Our results showed that mitochondrial transplantation prevented lysosomal membrane damage.

To evaluate the rat gender on mitochondrial transplantation, we tested both female and male rat mitochondria on the RPTCs affected by cisplatin. Some limited studies described the morphology and oxidative stress differences between male and female mitochondria in different organs (48). According to our findings, female mitochondria in the kidney of rat are more protective against ROS, MMP collapse, MDA, GSH depletion, lysosomal membrane damage, and caspase-3 activity than male mitochondria. In healthy conditions, female mitochondria are stronger than male mitochondria, and female mitochondria are also more resistant to hypoxic conditions (49), which is why it showed more grounded cell defensive impacts in our study.

### 5.1. Conclusions

**Figure 11.**
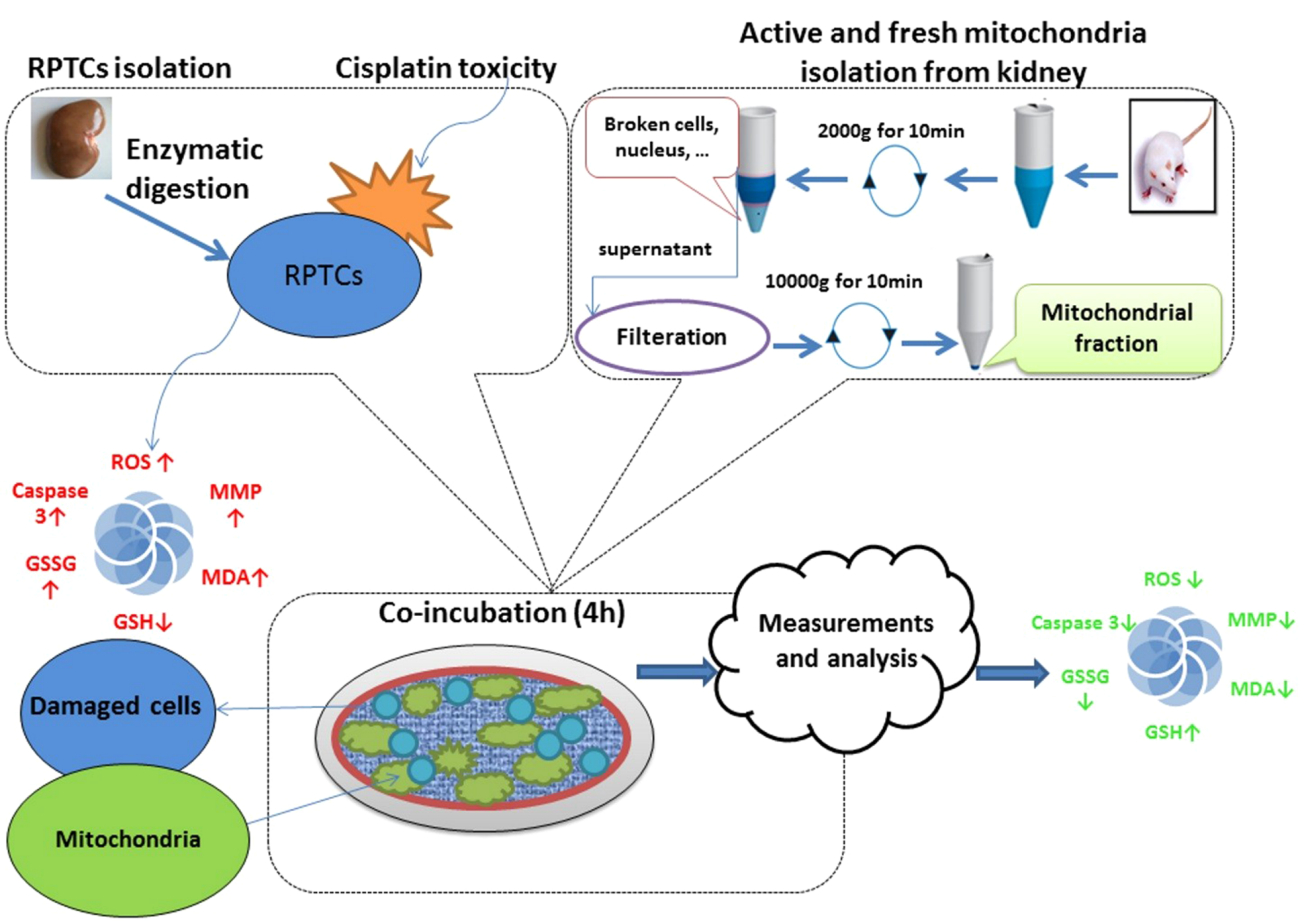
Schematic diagram of cisplatin-induced nephrotoxicity inhibition by mitochondrial transplantation. The healthy mitochondria were transferred to rat RPTCs, leading to ROS generation, oxidative injury, and caspase-3 activity amelioration.

In conclusion, as shown in Figure 11, we demonstrated that healthy mitochondria that successfully transferred into RPTCs reversed the cisplatin-induced mitochondrial dysfunction, oxidative stress, and cell death signaling pathway in rat RPTCs. Our findings open the potential for promising progress in treating mitochondrial diseases. Additionally, mitochondrial transplantation’s ability to reduce cisplatin-induced nephrotoxicity makes it a promising treatment for other chemical and drug-induced toxicities.

## Data Availability

The data presented in this study are available for readers upon request.
